# ERCP in Patients With Sickle Cell Disease: Diagnostic and Therapeutic Dilemmas

**DOI:** 10.4021/gr2010.03.177w

**Published:** 2010-03-20

**Authors:** Hussain Issa, Ali Al-Haddad, Ahmed H. Al-Salem

**Affiliations:** a Department of internal medicine, Qatif Central Hospital, Qatif, Saudi Arabia; b Current affiliation: King Fahad Specialist Hospital-Dammam, Saudi Arabia; c Current affiliation: Maternity and Children Hospital-Dammam, Saudi Arabia

**Keywords:** Sickle cell disease, Hepatobiliary, Cholestatic jaundice, ERCP

## Abstract

**Background:**

Cholestatic jaundice (CJ) in patients with sickle cell disease (SCD) poses diagnostic and therapeutic dilemmas. This is an evaluation of the role of ERCP in SCD.

**Methods:**

A total of 224 SCD patients with CJ had ERCP. The indications for ERCP were based on clinical and biochemical evidence of CJ and ultrasound findings.

**Results:**

The indications were: CJ only in79, CJ and dilated ducts in 103, and CJ and biliary stones in 42. The ERCP findings were: (A) For those with CJ only: ERCP was normal in 45, showed dilated ducts with no stones in 13, dilated ducts with stones in 16, normal CBD with a stone in 1; (B) For those with CJ, dilated ducts: ERCP was normal in 17, showed dilated ducts with stones in 47, dilated ducts without stones in 28, normal CBD with a stone in 1, a choledochoduodenal fistula in 2; (C) For those with CJ and duct stones: ERCP was normal in 2, showed dilated ducts with stones in 21, dilated ducts without stones in 14, normal CBD with a stone in 1.

**Conclusions:**

ERCP was unnecessary in a significant number (27%) of patients. This is especially so for those with CJ only (57%). These should be evaluated further prior to ERCP. There was also a significant number (19%) who had ES for duct dilatation without an obstruction. The reason for this dilatation is not known and the value of ES in this group needs to be investigated further.

## Introduction

Sickle cell disease which results from homzygous inheritance of hemoglobin S is one of the commonly inherited hemoglobinopathies worldwide. In the Eastern province of Saudi Arabia, SCD is common with a sickle cell trait frequency reaching up to 25% in some areas [1-3]. One of the common hepatobiliary complications of SCD is cholestatic jaundice which can be due to hepatic or extrahepatic causes [4-8]. Although these causes are different, they may however resemble each other clinically. This causes diagnostic and therapeutic difficulties. One of the diagnostic modalities for evaluating patients with cholestatic jaundice is ERCP. This is an evaluation of the role of ERCP in patients with SCD with emphasis on the diagnostic and therapeutic dilemmas.

## Patients and Methods

Over a period of 15 years (1993 - 2008), 224 patients with SCD underwent 240 ERCP procedures. Their medical records were reviewed and the following information was obtained: Age, sex, clinical features, indication for ERCP, ultrasound findings, ERCP findings, and therapeutic procedures at the time of ERCP, complications and outcome. All had an abdominal ultrasound and the indications for ERCP were based on clinical and biochemical evidence of cholestatic jaundice and ultrasound findings. Based on abdominal ultrasound findings, the patients were divided into three groups as follows: (1) those with normal ultrasound; (2) those with dilated bile ducts but no stones and (3) those with bile duct stones. All ERCPs were performed in the radiology department using Olympus TJF 240 or JF 260 side-viewing duodenoscope. This was done under general anesthesia with nasotracheal intubation for children less than 10 years old, and under sedation using meperdine (1 mg/kg) and diazepam (0.1 - 0.2 mg/kg) for those above 10 years of age. The ampulla of Vater was cannulated with tapered or regular catheters and the biliary ducts were deliberetly visualized under fluoroscopy using Hexabrix (320 mg diluted to 50%). Appropriate radiographs were obtained and where indicated sphincterotomy was performed using 5F sphincterotome (Olympus) and bile duct stones if found were extracted with a basket, balloon extractor or mechanical lithotrepter.

## Results

**Figure 1:**
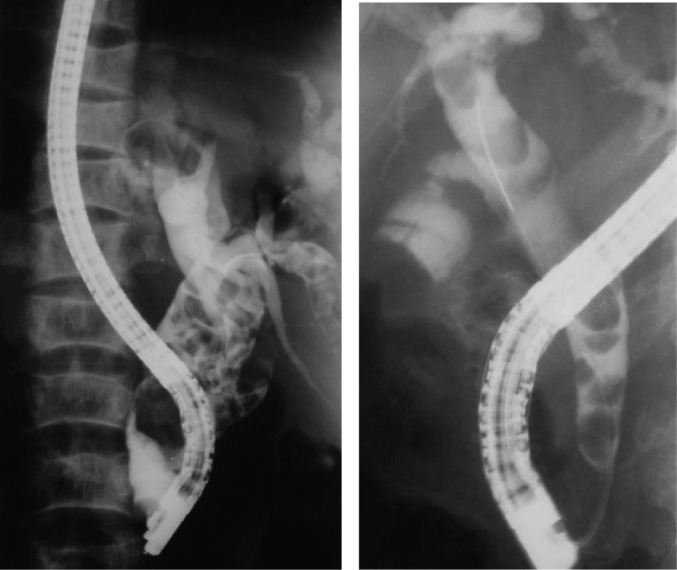
ERCP showing dilated bile ducts with stones in two different patients.

**Figure 2:**
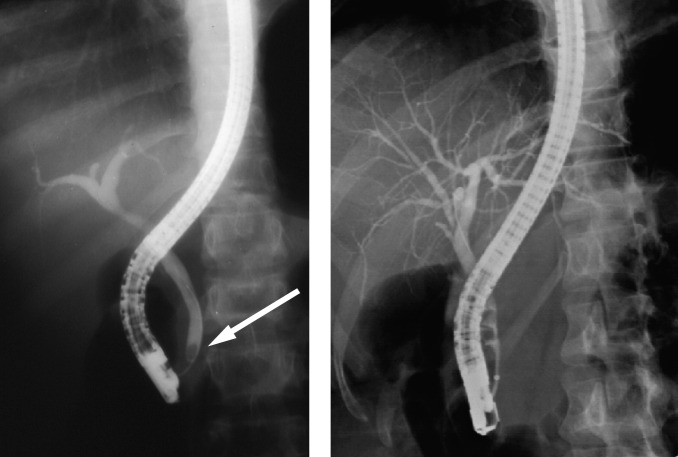
ERCP showing normal bile ducts with a stone in the lower CBD in one and multiple stones in the other.

**Figure 3:**
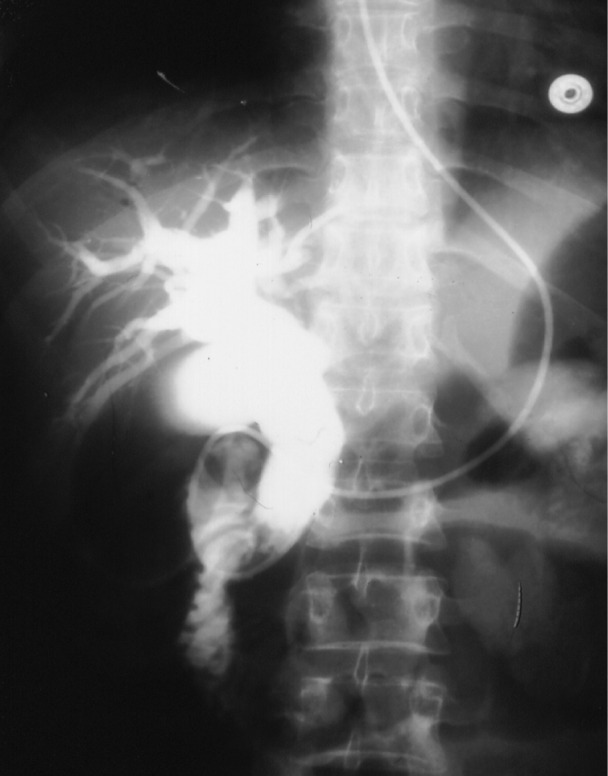
ERCP showing dilated bile ducts without an obstructive cause. Note the nasobiliary tube for drainage.

**Figure 4:**
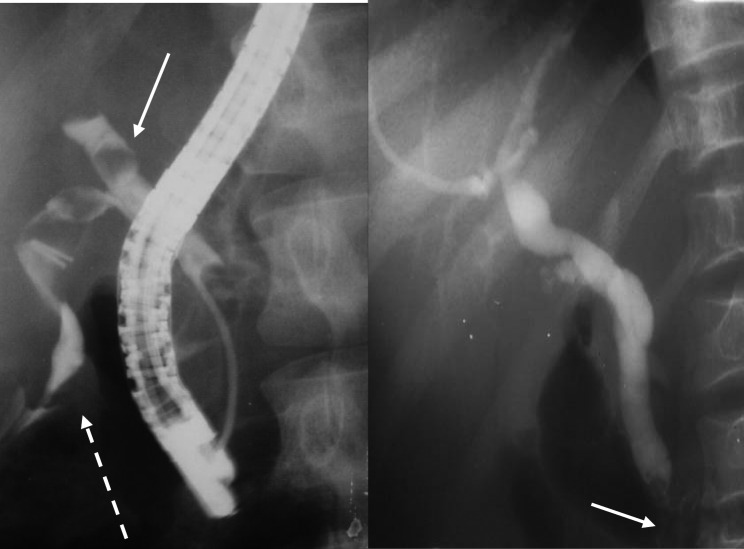
ERCP for two patients showing bile leak from the cystic duct (dotted arrow) and a stone in the bile ducts (solid arrow) following laparoscopic cholecystectomy in one and dilated bile ducts with stones in the lower CBD in another.

**Figure 5:**
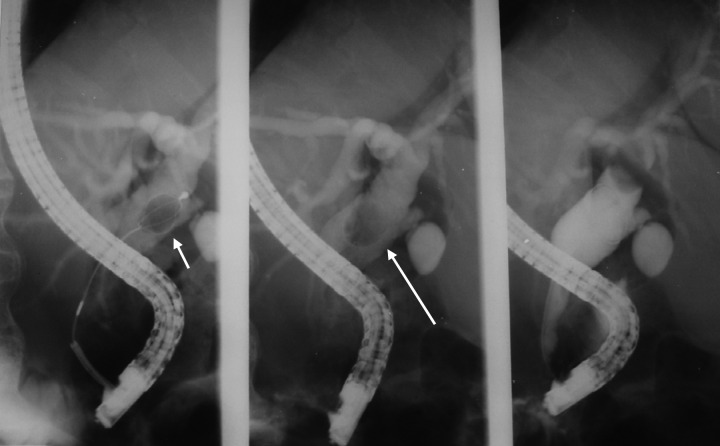
ERCP showing extraction of common bile duct stone.

A total of 224 patients with SCD underwent 240 ERCP procedures. There were 144 males and 80 females. Their mean age was 22.4 years (5 - 70 years), mean HbS was 76.8% (64.7% - 92.3%) and mean HbF was 20.4% (5.1% - 34.0%), mean total bilirubin was 22.4 mg/dl (5.5 mg/dl - 39.5 mg/dl), mean direct bilirubin was 13.4 mg/dl (4 mg/dl - 26.3 mg/dl), mean alkaline phosphatase was 486 I.U./ml (81 - 1189) (Normal: 50-136), mean ALT was 234.3 IU/ml (50 - 761) (Normal: 30-50) and mean AST was 206.3 IU/ml (63 - 317) (Normal: 15 - 37). The indications for ERCP were: cholestatic jaundice only in 97, cholestatic jaundice and dilated bile ducts on ultrasound in 103 and cholestatic jaundice and bile duct stones on ultrasound in 42. The ERCP findings in each of these three groups are shown in Table 1, Table 2 and Table 3. In those with cholestatic jaundice only, there was a group of 13 patients (16.5%) with dilated bile ducts without an obstructive cause and ERCP was normal in 45 (57%) of them. In this group, abdominal ultrasound failed to diagnose bile duct dilatation in 13 (16.5%) and failed to diagnose bile duct stones in 17 (21.5%), 16 of them had also bile duct dilatation (Fig. 1). In those with cholestatic jaundice and dilated bile ducts on ultrasound, there was a group of 28 patients (27.2%) with dilated bile ducts without an obstructive cause and ERCP was normal in 17 (16.5%) of them. In this group, abdominal ultrasound diagnosed bile duct dilation in 17 (16.5%) which were normal on ERCP and failed to diagnose bile duct stones in 48 (46.6%), one of them had a normal CBD (Fig. 2). In those with cholestatic jaundice and bile duct stones on ultrasound, there was a group of 14 patients (33.3%) with dilated bile ducts without an obstructive cause and ERCP was normal in 2 (4.8%) of them. In this group, abdominal ultrasound diagnosed bile duct stones in 2 (4.8%) which were normal on ERCP and another group of 14 ( 33.3% ) which had bile duct stones on ultrasound, had only dilatation of bile ducts but no stones on ERCP. In total 55 patients (24.6%) had dilated bile ducts without an obstructive cause and ERCP was normal in 64 (28.6%) patients (Fig. 3). Thirty-two had ERCP following cholecystectomy. Thirty of them presented with cholestatic jaundice and 2 had bile leak (Fig. 4). ERCP was normal in 7, showed dilated CBD with stones in 8, dilated CBD without stones in 5, dilated bile ducts with stones in 7, dilated bile ducts without stones in 2 and choledochoduodenal fistula in 1. In 2, ERCP showed bile leak from the cystic duct with a stone in the CBD in one of them. In 10 who had pancreatitis, ERCP was normal in 3, showed dilated CBD with a stone in 1, dilated CBD without stones in 3, one of them had an enlarged inflamed ampulla suggestive of recent stone passage, and 3 had dilated bile ducts with stones. In 12 who had cholangitis, ERCP showed dilated CBD with stones in 4, dilated CBD without stones in 1 and dilated bile ducts with stones in 7. The therapeutic procedures performed during ERCP are shown in Table 4. This included endoscopic sphincterotomy only in 42 out of the 55 patients (76.4%) who had dilated bile ducts without an obstructive cause. The remaining 13 were done early in the series and no endoscopic sphincterotomy was done. In total, 87 patients had bile duct stones and ERCP removed the stones in 95.4% of them (Fig. 5). There was no mortality. Four patients developed minor bleeding from the sphincterotomy site. This was controlled with local adrenaline injection. Eight (3.3%) developed transient mild pancreatitis.

**Table 1:** ERCP Findings in Patients With Cholestatic Jaundice Only (79 Patients)

Findings	No. of patients	%
Normal	45	57
Dilated CBD without stones	11	13.9
Dilated CBD with stones	10	12.7
Dilated CBD ducts with stones	6	7.6
Dilated bile ducts without stones	1	1.3
Dilated CBD without stones	1	1.3
Normal CBD with stones	1	1.3
Edematous inflamed papilla	4	5.1

**Table 2:** ERCP Findings in Patients With Cholestatic Jaundice and Dilated Bile Ducts on Ultrasound (103 Patients)

Findings	No. of patients	%
Normal	17	16.5
Dilated CBD without stones	17	16.5
Dilated CBD with stones	30	29.13
Dilated bile ducts without stones	11	10.7
Dilated bile ducts with stones	17	16.5
Normal CBD with a stone	1	0.98
Choledochduodenal fistula	2	1.94
Edematous inflamed papilla	8	7.8

**Table 3:** ERCP Findings in Patients With Cholestatic Jaundice and Bile Duct Stones (42 Patients)

Findings	No. of patients	%
Normal	2	4.8
Dilated CBD without stones	7	16.7
Dilated CBD with stones	14	33.3
Dilated bile ducts without stones	7	16.7
Dilated bile ducts with stones	7	16.7
Normal CBD with a stone	1	2.4
Edematous inflamed papilla	4	9.5

**Table 4:** Therapeutic Procedures During ERCP

Procedure	No. of patients	%
Endoscopic sphincterotomy	42	18.8
Endoscopic sphincterotomy and stone extraction	79	35.3
Insertion of biliary stent	8	3.6
Endoscopic sphincterotomy, mechanical lithortipsy and stone extraction	4	1.8
Insertion of a nasobiliary tube	4	1.8

## Discussion

Jaundice is one of the common manifestations of SCD. This can be caused by several diseases including intrahepatic and extrahepatic causes. One of the common intrahepatic causes is sickling of RBC within the liver sinuosoids which is also called hepatic crisis or hepatic sequestration (sickle cell hepatopathy) [4-8]. This can lead to cholestasis and a clinical picture that may resemble extrahepatic bile duct obstruction. Sickle cell intrahepatic cholestasis on the other hand is a more serious disease characterized by acute onset of hepatomegaly, hyperbilirubinemia, coagulopathy and acute liver failure [8]. Taking in consideration the high frequency of cholelithiasis and choledocholithiasis in patients with SCD, it is important to exclude these as a cause of cholestasis as soon as possible [9-12]. In a large number of patients this distinction can be made based on clinical, biochemical and ultrasound evaluation. This however is not the case always and further more invasive investigation may be needed including ERCP. Abdominal ultrasound is a simple, and non invasive investigation and it is valuable in detecting gallbladder stones and dilatation of the extra and intrahepatic bile ducts, but bile duct stones may be missed in as much as 60% of patients [13]. In our series, abdominal ultrasound failed to diagnose bile duct stones in 29% of the patients. Abdominal ultrasound is also an operator dependent. Considering the high incidence of bile duct stones in patients with SCD, it is important to exclude these as a cause of cholestatic jaundice whether pre or postcholecystectomy. This is specially so in the era of laparoscopic cholecystectomy [11-14]. We found ERCP valuable in this regard. Sequential endoscopic sphincterotomy and stone extraction followed by laparoscopic cholecystectomy is beneficial in these patients [14]. ERCP however, is more invasive than an abdominal ultrasound, but it provides direct visualization of the biliary tree as well as demonstrating the site and nature of the obstructive lesion. Add to this, the valuable therapeutic interventions which can be done via ERCP [15]. This was the case in our series where we found ERCP valuable as a diagnostic and therapeutic procedure in patients with SCD. Our series however, is a unique group of patients with a large number of them having bile duct stones. Eighty-seven (38.8%) of our patients had bile duct stones and ERCP was valuable in removing these stones in 83 of them (95.4%). This is also the case for those who already had undergone cholecystectomy. Fifteen of our 32 post-cholecystectomy patients who had ERCP had documented bile duct stones which were removed via ERCP. It is difficult to ascertain the origin of these bile duct stones whether they were primary left over from before at the time of cholecystectomy or secondary, formed later on as a sequalae of SCD.

ERCP was however unnecessary in a significant number of our patients (27%). This is specially so for those who presented with cholestatic jaundice only (57%). These patients most likely had cholestatic jaundice secondary to intrahepatic sickling of RBC or represent a benign variant of intrahepatic cholestasis or a form of what is called benign hyperbilirubinemia [5]. To obviate this, these patients should be investigated further prior to ERCP including endoscopic ultrasound and magnetic resonance cholangiopancreatography. These investigations however are not readily available and a period of conservative management including observation, hydration and simple or exchange blood transfusion.

Although ERCP is also valuable as a therapeutic procedure, a significant number of our patients with bile duct dilatation without an obstructive cause had endoscopic sphincterotomy (76.4%). The etiology of this dilation is not known but most likely this is a form of sickle cell cholangiopathy [16, 17]. It is well known that patients with SCD are more prone to have biliary sludge as well as bile duct stones. This is specially so in the presence of dilated bile ducts. We feel that endoscopic sphincterotomy in those with bile duct dilatation should be beneficial in preventing future development of bile duct stones. This however needs to be evaluated further.

In conclusion, ERCP is valuable in patients with SCD both as a diagnostic and therapeutic procedure. Patients with cholestatic jaundice only without evidence of bile duct dilatation of stones on ultrasound should be evaluated further prior to ERCP. SCD patients with bile duct dilatation without an obstructive cause may benefit from endoscopic sphincterotomy. This however needs to be evaluated further.
